# Cross-fostering does not alter the neurochemistry or behavior of spontaneously hypertensive rats

**DOI:** 10.1186/1744-9081-5-24

**Published:** 2009-06-23

**Authors:** Fleur M Howells, Leander Bindewald, Vivienne A Russell

**Affiliations:** 1 Neuroscience Laboratory, Division of Physiology, Department of Human Biology, Faculty of Health Sciences, University of Cape Town, Observatory, 7925, South Africa

## Abstract

**Background:**

Attention-deficit/hyperactivity disorder (ADHD) is a highly heritable developmental disorder resulting from complex gene-gene and gene-environment interactions. The most widely used animal model, the spontaneously hypertensive rat (SHR), displays the major symptoms of ADHD (deficits in attention, impulsivity and hyperactivity) and has a disturbance in the noradrenergic system when compared to control Wistar-Kyoto rats (WKY). The aim of the present study was to determine whether the ADHD-like characteristics of SHR were purely genetically determined or dependent on the gene-environment interaction provided by the SHR dam.

**Methods:**

SHR/NCrl (Charles River, USA), WKY/NCrl (Charles River, USA) and Sprague Dawley rats (SD/Hsd, Harlan, UK) were bred at the University of Cape Town. Rat pups were cross-fostered on postnatal day 2 (PND 2). Control rats remained with their birth mothers to serve as a reference for their particular strain phenotype. Behavior in the open-field and the elevated-plus maze was assessed between PND 29 and 33. Two days later, rats were decapitated and glutamate-stimulated release of [^3^H]norepinephrine was determined in prefrontal cortex and hippocampal slices.

**Results:**

There was no significant effect of "strain of dam" but there was a significant effect of "pup strain" on all parameters investigated. SHR pups travelled a greater distance in the open field, spent a longer period of time in the inner zone and entered the inner zone of the open-field more frequently than SD or WKY. SD were more active than WKY in the open-field. WKY took longer to enter the inner zone than SHR or SD. In the elevated-plus maze, SHR spent less time in the closed arms, more time in the open arms and entered the open arms more frequently than SD or WKY. There was no difference between WKY and SD behavior in the elevated-plus maze. SHR released significantly more [^3^H]norepinephrine in response to glutamate than SD or WKY in both hippocampus and prefrontal cortex while SD prefrontal cortex released more [^3^H]norepinephrine than WKY. SHR were resilient, cross-fostering did not reduce their ADHD-like behavior or change their neurochemistry. Cross-fostering of SD pups onto SHR or WKY dams increased their exploratory behavior without altering their anxiety-like behavior.

**Conclusion:**

The ADHD-like behavior of SHR and their neurochemistry is genetically determined and not dependent on nurturing by SHR dams. The similarity between WKY and SD supports the continued use of WKY as a control for SHR and suggests that SD may be a useful additional reference strain for SHR. The fact that SD behaved similarly to WKY in the elevated-plus maze argues against the use of WKY as a model for anxiety-like disorders.

## Background

Attention-deficit/hyperactivity disorder (ADHD) is a heterogeneous disorder resulting from complex gene-gene and gene-environment interactions which give rise to variable expression of the defining symptoms of impaired sustained attention, impulsivity and hyperactivity [1-5]. ADHD is highly heritable [4-6]. A genome-wide association scan of quantitative traits for ADHD identified a wide range of genes implicating the GABA transporter, sodium/hydrogen exchanger, noradrenergic, serotonergic, dopaminergic and nicotinic receptors as well as genes that encode proteins involved in the synthesis and transport of norepinephrine, strongly implicating the noradrenergic system in ADHD [6]. In addition to playing a critical role in the regulation of attention and arousal, the noradrenergic system promotes secondary behaviors such as vigilance, exploratory activity, and behavioral flexibility, disturbance of which could give rise to symptoms of ADHD [7-10]. To explain the heterogeneous nature of ADHD, it has been suggested that different combinations of genetic and environmental factors may be required to produce individual clusters of behavioral symptoms [11-15]. Environmental risk factors that contribute significantly to ADHD, include prenatal exposure to drugs such as alcohol and nicotine, obstetric complications, head injury and psychosocial adversity, suggesting that the early postnatal environment may be an important contributory factor [16-18].

Neurophysiological and imaging studies have shown that ADHD is associated with alterations in several brain structures involved in the regulation of behavior, including the prefrontal cortex and its connections to the striatum, parietal cortex and cerebellum [19-25]. The prefrontal cortex is important for sustaining attention over a delay, inhibiting distraction and dividing attention, while the parietal cortex is essential for perception and the allocation of attentional resources [25]. There is compelling evidence that both noradrenergic and dopaminergic systems are altered in ADHD, norepinephrine enhances neural signalling by acting on α_2A_-adrenoceptors in prefrontal cortex to strengthen functional connectivity in neural networks while dopamine decreases "noise" through modest levels of DRD1 activation [25,26]. Deficient norepinephrine or dopamine modulation of the strength of connections in sensorimotor networks may impair or delay their maturation which is thought to occur in patients with ADHD as evidenced by increased latency of evoked potentials in the auditory and visual systems, increased theta relative to alpha or beta power in the EEG and reduced coherence of EEG waveforms between the cerebral hemispheres [27-31].

Development of the brain follows a precise genetically determined programme that is subject to modification by the environment [32]. Sensory stimulation and experience affect norepinephrine and dopamine release and initially increase the number of synaptic connections between neurons [32]. Dendritic pruning and synapse elimination produce more efficient neural circuits that continue to be remodelled throughout life [32]. Any disruption of this process can result in impaired brain function. Consistent with ADHD being a developmental disorder, rat models of ADHD are either genetically determined or require pre- or postnatal intervention [33,34]. Early postnatal conditions can be manipulated experimentally by altering the maternal environment of the pups. Cross-fostering and maternal separation are widely used to study the influence of early postnatal environment on rat pups [35-39].

Similar to children with ADHD, there is considerable evidence to suggest that disturbances in the noradrenergic system may contribute to the development of ADHD-like behavior in a widely used rat model of ADHD, the spontaneously hypertensive rat (SHR). SHR display the major symptoms of ADHD such as deficits in attention, impulsivity and hyperactivity when compared to Wistar-Kyoto rats (WKY), the strain from which they were derived, as well as other rat strains [40-44]. SHR have been shown to have poor autoreceptor-mediated feedback control of norepinephrine release and increased glutamate-stimulated release of norepinephrine from terminals of locus coeruleus neurons, in addition to disturbances in the dopaminergic system [45-51]. However, the use of WKY as a control for SHR has recently been questioned because of instability in its behavioral characteristics [44]. WKY obtained from certain suppliers have been suggested to model the inattentive subtype of ADHD, while other studies have suggested that WKY may be used as a model of anxiety or depression [44,52-54]. Sprague Dawley rats (SD) were therefore included in the present study as an additional control for SHR.

The aim of the present study was to investigate whether the early postnatal environment determines behavioral and neurochemical outcomes in SHR, WKY and SD rat strains. Rat pups were subjected to cross-fostering and the effects on their exploratory behavior and anxiety-like behavior determined in an open-field apparatus and elevated-plus maze. These studies were performed when the rats were 4 weeks of age since SHR are hyperactive at this age and have not yet developed signs of hypertension [54-56]. Glutamate-stimulated release of norepinephrine in hippocampal and prefrontal cortical slices was also measured to indicate whether change in the early postnatal environment had altered glutamate regulation of the locus coeruleus noradrenergic system, since this is altered in SHR and these brain areas are involved in processing and responding to sensory input from the environment during the early stages of development. The hypothesis tested in this study was that the ADHD-like characteristics of SHR, specifically hyperactivity and lack of anxiety, were purely genetically determined and not the result of interaction between the pup and the environment provided by the SHR dam.

## Methods

### Animals

SHR/NCrl (Charles River Laboratories, USA), WKY/NCrl (Charles River Laboratories, USA) and SD/Nsd (Harlan Laboratories, UK) were bred by the University of Cape Town Animal Unit. Rats obtained from the Animal Unit were housed in plastic cages with sawdust bedding in a 12 hr light/dark cycle (lights on from 06h00 to 18h00). Food and water were provided *ad libitum *and the temperature was maintained between 21 and 23°C. The rats were transferred to a clean cage three times per week. All experiments were approved by the Research Ethics Committee of the University of Cape Town.

### Cross-fostering protocol

Two or three SHR, WKY or SD adult female rats were placed with a male (harem mating) of the same strain for four days (the duration of the oestrous cycle of the rat, thereby increasing the likelihood of successful mating). The females were housed individually when signs of pregnancy became evident. The pregnant dams were monitored daily and the date of birth of pups (PND 0) was noted. All pups were treated identically to avoid the confounding effects of handling. On PND 2, pups were sexed and litters were culled to eight. All pups were transferred to clean cages. Each dam was gently placed in the cage that held the litter that it would rear (either its own pups or cross-fostered pups). Cross-fostering took place on PND 2 to minimize the possibility of cannibalism, which could possibly occur as a result of (1) human handling at too early an age, (2) insufficient time allowed for grooming and removal of traces of delivery, and (3) heightened maternal sensitivity during the first few PNDs. This method of cross-fostering on PND 2 was found to be highly successful in all three rat strains. Pups born in one litter were cross-fostered as one litter. The litters were not mixed at any stage. Control rats remained with their birth mothers, to closely mimic normal rats. Litters were culled to 8 pups per litter. Litters of less than 5 pups were not included in the study. On PND 21 the rats were weaned and paired with a litter mate of the same strain and rearing condition. Rat pups (n = 10 to 15 per group, 5 to 6 litters, 2 to 4 rats from each litter) were assessed for their behavior in the open-field and the elevated-plus maze between PND 29 and 33. Two days after the behavioral recordings, the rats were decapitated to determine glutamate-stimulated release of [^3^H]norepinephrine in prefrontal cortex and hippocampal slices.

### Maternal separation protocol

A second model frequently used to study the effects of altered maternal environment on brain development makes use of chronic maternal separation (3 h per day for 14 days) which causes long-lasting changes in brain function [57]. SD dams were harem mated as described above. Females were housed individually when signs of pregnancy became evident. The date of birth (PND 0) of the pups was noted. On PND 2 litters were culled to 8 pups. From PND 2 through to PND 14 the dams were removed from the litters (the pups were not handled) for 3 h per day. The separation occurred between 09h00 and 12h00. Cages containing the pups were transferred to a separate room where the temperature was maintained at 31°C to prevent hypothermia. At 12h00 the cages with pups were returned to the communal rat room and maternal dams were returned to their pups. Rats belonging to the control group were raised normally. Pups were weaned in litters on PND 21. From PND 30 to PND 35, rats were housed in pairs. On PND35 rats were decapitated to determine glutamate-stimulated release of [^3^H]norepinephrine in prefrontal cortex and hippocampal slices.

### Behavioral measures

Between PND 29 and 33 behavior in the open-field and elevated-plus maze was assessed. The rats were taken to a room adjacent to the behavioral room at least 1 h prior to behavioral recording which took place between 10h00 and 14h00. Since novelty is a major contributor to behavior in the open-field, this test was performed initially, at least 2 h prior to the elevated-plus maze. Illumination of the behavioral room was 50 lux to encourage exploration of the open-field apparatus [37]. The elevated-plus maze provided a robust measure of anxiety-like behavior which was not sensitive to changes in illumination [58]. The rats' behavior was recorded with video cameras and analyzed with Ethovision software (version 3.1, Noldus Information Technology, Wageningen, Netherlands). Each apparatus was cleaned with 20% ethanol between rat recordings.

### Open-field behavior

The inner zone (0.70 m × 0.70 m) of the open-field (1.0 m × 1.0 m black wooden box with 0.5 m high walls) was demarcated with a white line. Each rat was individually placed in the outer zone of the open-field facing into a corner, parallel to a wall. The positioning of the rat within the open-field in this manner was to limit locomotion resulting from the stress of handling and placement into the novel environment, which may have yielded a false locomotor response. The rat's behavior was recorded for 15 min. Parameters analysed in the open-field apparatus were (a) total distance travelled, (b) time taken to enter the inner zone, (c) number of entries into the inner zone, and (d) time spent in the inner zone [37].

### Elevated-plus maze behavior

Each rat's behavior in the elevated-plus maze (1.0 m × 1.0 m black plastic plus-shaped apparatus, raised above the ground by 0.5 m) was recorded for 5 min after a 2-h period of rest after exploring the open-field apparatus. Rats were placed in the centre of the elevated-plus maze facing an open arm. Parameters analyzed in the elevated-plus maze included (a) time spent in the closed arms, (b) time spent in the open arms, and (c) number of entries into the open arms [58].

### Neurochemistry

Two days after the behavioral measures (PND31 to PND35), rats were transferred to a room adjacent to the laboratory 1 h prior to decapitation. Rats were killed by decapitation in their light cycle, between 09h00 and 12h00. Their brains were rapidly removed and submerged in ice-cold Krebs buffer (NaCl 118 mM, KCl 4.7 mM, NaH_2_PO_4_.H_2_0 1.0 mM, MgCl_2_.6H_2_O 1.2 mM, NaHCO_3 _23 mM, D-Glucose 11 mM, EDTA 37.6 μM and CaCl_2_.H_2_O 1.3 mM) and aerated with carbogen (95% O_2_/5% CO_2_) for 15 min as previously described [48,49,51].

Prefrontal cortex was dissected from three anterior 0.9 mm coronal brain sections and chopped into 0.3 mm by 0.3 mm slices with a McIlwain tissue chopper. Hippocampi were removed and similarly chopped into 0.3 mm by 0.3 mm slices. The tissue slices were transferred to ice-cold Krebs buffer (1 ml) containing ascorbic acid (5.7 mM, to reduce free radical damage) and transferred to a waterbath maintained at 37°C. After 10 min, radioactively labelled norepinephrine (2.67 μl, 1-[7,8-^3^H]norepinephrine, 37 MBq/ml, 1.0 mCi/ml, Amersham International, UK) was added and incubated with the tissue for 15 min to allow uptake of [^3^H]norepinephrine by vesicles in noradrenergic axons within the tissue slices. After 15 min, the supernatant was removed and fresh Krebs buffer was added to the tissue. The slices were transferred to superfusion chambers and perfused with Krebs buffer for 1 h. Two 5-min baseline fractions of eluate were collected from the columns. Upon initiation of collection of the third fraction, the inlet tubes of the superfusion columns were transferred to a 1 mM glutamate-containing Krebs buffer solution. The inlet tubes were kept in the glutamate-containing Krebs buffer solution for 1 min, and then returned to the Krebs buffer solution for the remaining 4 min of the fraction. This fraction served as the glutamate-stimulated fraction. An additional 5-min baseline fraction and a final fraction were collected. The brain slices were removed from the superfusion columns, 1 ml 0.1 M NaOH was added and radioactivity remaining in the slices determined.

### Calculation of glutamate-stimulated release of [^3^H]norepinephrine

The radioactivity in baseline and stimulation fractions as well as radioactivity in the brain slices at the end of the experiment was analyzed using a Packard 1900 CA TRI-CARB liquid scintillation analyzer. To determine glutamate-stimulated release relative to baseline, release of radioactivity was calculated as a fraction of the total amount of radioactivity present in the slices at the time of release of that 5-min fraction, and baseline fractional release was subtracted from the stimulation fractional release, to obtain glutamate-stimulated release of radioactivity.

### Statistics

A two-way analysis of variance (ANOVA) with factors "strain of dam" and "pup strain" was applied to the data. This was followed by Tukey's HSD post-hoc test where appropriate, using Statistica 8 software. Results are expressed as mean ± SEM.

## Results

**Figure 1:**
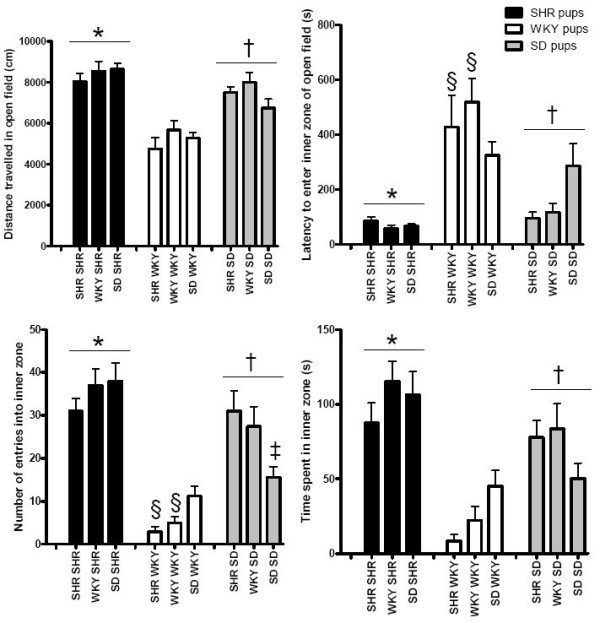
**Effect of cross-fostering on behavior in the open-field apparatus**. *SHR pups (n = 12 – 14) travelled a greater distance (two-way ANOVA, significant effect of "pup strain", F_(2,100) _= 46, P < 0.0001, post-hoc Tukey's HSD test, P < 0.01), spent a longer period of time in the inner zone (two-way ANOVA, F_(2,100) _= 31, P < 0.0001, post-hoc Tukey's HSD test, P < 0.005) and entered the inner zone more frequently (two-way ANOVA, F_(2,100) _= 52, P < 0.0001, post-hoc Tukey's HSD test, P < 0.005) than WKY (n = 11 – 12) and SD (n = 10 – 15). SHR also entered the inner zone more rapidly than WKY (two-way ANOVA, F_(2,100) _= 30, P < 0.0001, post-hoc Tukey's HSD test, P < 0.0005). ^†^SD pups travelled a greater distance (P < 0.0005), spent more time in the inner zone (P < 0.0005) and entered the inner zone more frequently (P < 0.0005) than WKY pups. SD entered the inner zone more rapidly than WKY (P < 0.0005). ^§^Significantly different from SD reared by SHR or WKY (two-way ANOVA, significant interaction between "strain of dam" and "pup strain", F_(4,100) _> 2.8, P < 0.05, post-hoc Tukey's HSD test, P < 0.005). ‡Significantly less than SD reared by SHR, and SHR reared by WKY or SD (two-way ANOVA, significant dam*pup interaction, F_(4,100) _= 4.0, P < 0.005, post-hoc Tukey's HSD test, P < 0.005).

**Figure 2:**
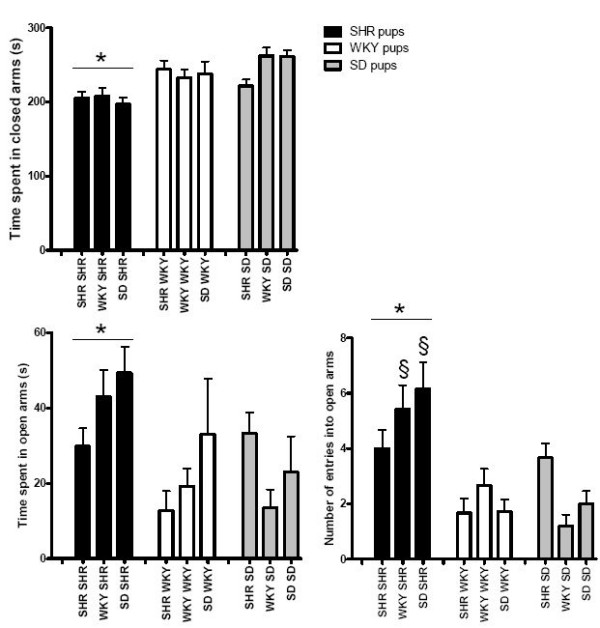
**Effect of cross-fostering on behavior in the elevated-plus maze**. *SHR pups (n = 12 – 14) spent less time in the closed arms (two-way ANOVA, significant effect of "pup strain" F_(2,100) _= 13.9, P < 0.0001, post-hoc Tukey's HSD test, P < 0.001), more time in the open arms (two-way ANOVA, F_(2,100) _= 6.0, P < 0.005, post-hoc Tukey's HSD test, P < 0.05) and entered the open arms more frequently (two-way ANOVA, F_(2,100) _= 22, P < 0.0001, post-hoc Tukey's HSD test, P < 0.0005) than WKY (n = 11 – 12) and SD (n = 10 – 15). ^§^Significantly greater than SD reared by WKY or SD (two-way ANOVA, significant interaction between "strain of dam" and "pup strain", F_(4,100) _= 3.7, P < 0.01, post-hoc Tukey's HSD test, P < 0.005).

**Figure 3:**
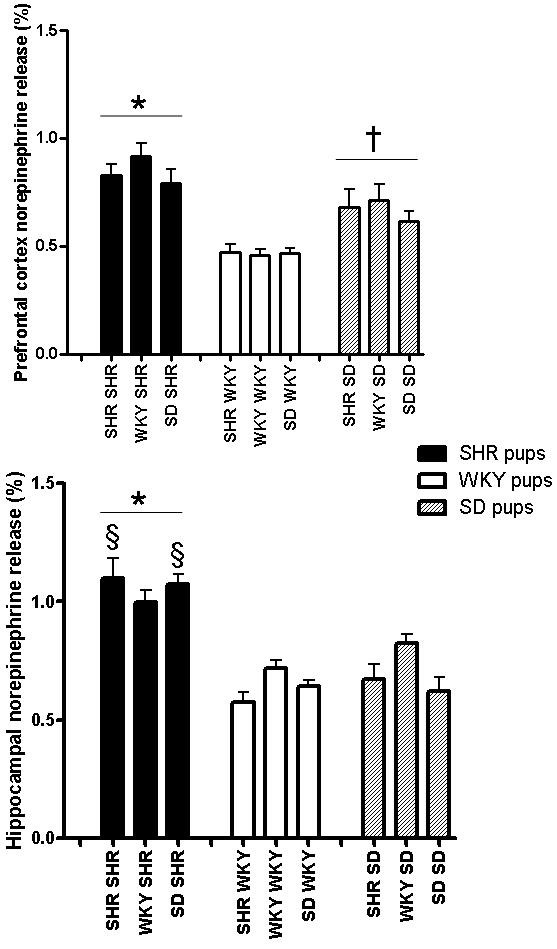
**Effect of cross-fostering on [^3^H]norepinephrine release in prefrontal cortex and hippocampus of SHR (n = 12 – 14), WKY (n = 11 – 12) and SD (n = 10 – 15)**. *SHR released significantly more [^3^H]norepinephrine in response to glutamate than SD or WKY in hippocampus (two-way ANOVA, significant effect of "pup strain" F_(2,100) _= 52, P < 0.0001, post-hoc Tukey's HSD test, P < 0.0005) and prefrontal cortex (two-way ANOVA, significant effect of "pup strain" F_(2,100) _= 32, P < 0.0001, post-hoc Tukey's HSD test, P < 0.001). ^†^SD pups released more [^3^H]norepinephrine than WKY in prefrontal cortex (P < 0.0005). ^§^Significantly greater than SD reared by SHR or SD dams (two-way ANOVA, significant interaction between "strain of dam" and "pup strain", F_(4,100) _= 2.6, P < 0.05, post-hoc Tukey's HSD test, P < 0.0005).

A two-way ANOVA revealed a significant effect of "pup strain" (F_(2,100) _> 6, P < 0.005) for all parameters investigated and a significant interaction between "strain of dam" and "pup strain" for various behavioral parameters including latency to enter the inner zone of the open-field (F_(4,100) _= 2.81, P < 0.05, Figure 1), frequency of entries into the inner zone of the open-field (F_(4,100) _= 3.96, P < 0.005, Figure 1) and frequency of entries into the open arms of the elevated-plus maze (F_(4,100) _= 3.67, P < 0.01, Figure 2), as well as glutamate-stimulated release of [^3^H]norepinephrine in rat pup hippocampus (F_(4,100) _= 2.55, P < 0.05, Figure 3). There was no significant effect of "strain of dam" (F_(2,100) _< 2.1, P > 0.1).

### Pup strain differences

In the open-field, SHR pups travelled a greater distance (P < 0.01), spent a longer period of time in the inner zone (P < 0.005) and entered the inner zone more frequently (P < 0.005) than SD and WKY (Figure 1). SD pups travelled a greater distance (P < 0.0005), spent more time in the inner zone (P < 0.0005) and entered the inner zone more frequently (P < 0.0005) than WKY pups (Figure 1). WKY took longer to enter the inner zone (P < 0.0005) than SHR or SD pups.

In the elevated-plus maze, SHR spent less time in the closed arms (P < 0.001), more time in the open arms (P < 0.05) and entered the open arms more frequently (P < 0.0005) than SD or WKY pups (Figure 2). There was no difference between WKY and SD behavior in the elevated-plus maze.

Post-hoc Tukey's HSD test revealed that SHR released significantly more [^3^H]norepinephrine in response to glutamate than SD or WKY pups in both hippocampus (P < 0.0005) and prefrontal cortex (P < 0.001) while SD prefrontal cortex released more [^3^H]norepinephrine than WKY (P < 0.0005, Figure 3).

### Interactions between pup strain and strain of dam

Cross-fostering appeared to increase the exploratory behavior of SD pups in the open-field. SD pups cross-fostered onto WKY or SHR dams displayed shorter latencies to enter the inner zone of the open-field and entered the inner zone of the open-field more frequently than WKY pups reared by WKY or SHR dams (P < 0.005) while SD pups reared by SD dams were not significantly different from WKY pups (P > 0.1, Figure 1).

SD pups reared by SD dams entered the inner zone of the open-field less frequently than SD pups cross-fostered onto SHR dams (P < 0.05) and SHR pups reared by SD or WKY dams (P < 0.005). SD pups reared by SHR dams entered the inner zone as frequently as SHR pups reared by SHR dams. SHR dams appeared to have opposite effects on SHR and SD pups in the open-field.

SHR pups cross-fostered onto WKY or SD dams entered the open arms of the elevated-plus maze more frequently than SD pups reared by WKY or SD dams (P < 0.005, Figure 2). SD pups cross-fostered onto SHR dams behaved similar to SHR pups reared by SHR dams. SHR dams tended to have opposite effects on SD and SHR behavior in the elevated-plus maze.

SHR reared by either SHR or SD dams released more hippocampal [^3^H]norepinephrine in response to glutamate than SD pups reared by either SHR or SD dams (P < 0.0005, Figure 3). SHR pups reared by WKY dams did not differ from SD pups reared by WKY dams (P > 0.3). Cross-fostering onto WKY dams had opposite effects on glutamate-stimulated release of [^3^H]norepinephrine in hippocampus of SD and SHR.

### Maternal separation

Maternal separation did not effect glutamate-stimulated release of [^3^H]norepinephrine from either hippocampal or prefrontal cortical slices of SD rats (ANOVA, P > 0.3).

## Discussion

The strain of the dam did not alter the behavior of SHR in the open-field and elevated-plus maze. It also did not affect glutamate release in hippocampus or prefrontal cortex of SHR, WKY or SD rats. However, there was a significant effect of "pup strain" on all parameters measured and a significant interaction between "strain of dam" and "pup strain" in several behavioral parameters related to exploratory activity, namely latency to enter the inner zone of the open-field, frequency of entries into the inner zone of the open-field and frequency of entries into the open arms of the elevated-plus maze as well as glutamate-stimulated release of [^3^H]NE in hippocampal slices. There was no interaction between the "strain of the dam" and "pup strain" in anxiety-like behaviors, namely, time spent in the inner zone of the open-field and time spent in the open arms of the elevated-plus maze, suggesting no effect on anxiety-like behavior. There was also no difference between the strains in the effect of maternal environment on distance travelled by the pups in the open-field, suggesting no effect on locomotor activity. SHR dams tended to decrease hippocampal release of NE in response to glutamate in SD and WKY relative to rats reared by WKY dams while SHR did not show this trend. These changes in glutamate regulation of NE release were accompanied by an increase in exploratory behavior in SD reared by SHR dams evidenced by a decrease in latency to enter the inner zone of the open-field and an increase in frequency of entries into the inner zone of the open-field and open arms of the elevated-plus maze. SD rats cross-fostered onto WKY dams also showed increased exploratory behavior when compared to SD rats reared by SD dams, reflected by decreased latency to enter the inner zone and increased frequency of entry into the inner zone of the open-field but not the elevated-plus maze, possibly because of the increased element of anxiety caused by the elevation of the plus maze. In contrast, SHR were not affected by cross-fostering onto SD or WKY dams, their phenotype did not appear to be affected by maternal environmental changes in the early stages of development.

Of the three rat strains, SD appeared to be the most severely affected by cross-fostering significantly increasing their exploratory behavior. The effect of cross-fostering was greater in SD possibly because SD rats differ from the two Wistar-Kyoto derived rat strains that are genetically more closely related to each other than to the SD rat strain. It was therefore decided to investigate whether a second model of altered postnatal environment would cause neurochemical changes in the brains of SD rats. The mild postnatal stress of maternal separation has been shown to induce anxiety-like behavior in SD rats causing long-term changes in neural circuits that control behavior and reactivity to stress [35,57]. However, when SD rats were subjected to chronic maternal separation there was no change in glutamate-stimulated release of norepinephrine in prefrontal cortex or hippocampus of SD rats, suggesting that these neuronal circuits were not affected by maternal separation stress.

In agreement with previous reports, SHR displayed increased locomotor activity and increased exploratory behavior when compared to WKY in the open field, evidenced by reduced time to enter the inner zone of the open-field, greater frequency of entries into the inner zone and increased time spent in the inner zone of the open-field [59-63]. SD were intermediate between SHR and WKY in terms of exploratory behavior in the open field and hippocampal release of norepinephrine.

SHR displayed the ADHD-like characteristic of decreased anxiety-like behavior in the elevated-plus maze relative to both WKY and SD. SHR pups spent less time in the closed arms, more time in the open arms and entered the open arms more frequently than WKY and SD pups. The difference in the pattern of behavior of SHR, SD and WKY observed in the elevated-plus maze may be due to the increased anxiety-inducing effect of the elevated maze relative to the open-field. The fact that there were no differences between WKY and SD behavior in the elevated-plus maze suggests that WKY are not abnormally anxious and supports the use of WKY as a control for SHR. The results of the present study further support the use of SD as an additional reference strain for SHR.

An interesting finding was the pattern of glutamate-stimulated release of norepinephrine in prefrontal cortex (but not hippocampus) of SHR, SD and WKY which was similar to the pattern of their behavior in the open-field (but not the elevated-plus maze). SD were intermediate between SHR and WKY in terms of norepinephrine release in response to glutamate and also in their exploratory behavior in the open-field. It is possible that glutamate-stimulated release of norepinephrine in the prefrontal cortex reflects activity of working memory and that behavior in the open-field involves activation of working memory in a novel environment. Increased neural activity would lead to glutamate release in the prefrontal cortex. One of its effects is to stimulate astrocytes to release lactate as the preferred substrate for ATP production by neurons undergoing rapid and/or sustained firing [64,65]. Glutamate also stimulates norepinephrine release [49,66]. Norepinephrine is known to stimulate glycolysis and lactate production in astrocytes [64]. It is therefore not unlikely that glutamate-stimulated release of norepinephrine in prefrontal cortex is upregulated in rats that display increased exploratory behavior.

### Limitations

Limitations to interpretation of the results in terms of a rat model for ADHD include the fact that the SHR begin to develop hypertension from 4-weeks of age which is a confounding factor for most behavioral studies that have been used to characterize the ADHD-like behavior of SHR. This complication was avoided in the present study by performing the experiments when the rats were 4 weeks of age since SHR are hyperactive at this age and have not yet developed signs of hypertension. Another concern that has emerged in recent times, is the failure to demonstrate "impulsivity" in SHR [44]. Nevertheless, Sagvolden and colleagues have shown that SHR provide a robust model for ADHD-like hyperactivity and failure to learn complex tasks [42,44]. Perhaps a more severe limitation to studies of the SHR rat model of ADHD lies in the instability in the behavioral characteristics of its normotensive control rat, the WKY [44]. WKY obtained from certain suppliers have been suggested to model the inattentive subtype of ADHD, while other studies have suggested that WKY may be used as a model of anxiety or depression [44,52-54]. Although not ideal, SD were included in the present study as an additional control for SHR.

## Conclusion

Cross-fostering did not alter the behavioral characteristics of SHR, suggesting that the ADHD-like behavior of SHR is genetically determined and not the result of gene-environment interactions provided by SHR dams. Cross-fostering of SD pups onto SHR or WKY dams increased exploratory behavior without altering their anxiety-like behavior. The evidence presented in this paper provides support for the use of WKY as a control strain for SHR. The fact that the behavior of SD was similar to WKY in the elevated-plus maze argues against the use of WKY as a model for anxiety-like disorders.

In general, SHR and WKY represent the extreme ends of behavioral variation in tests of anxiety, locomotor activity and exploratory behavior, consistent with the use of SHR as an animal model for ADHD and also with the use of WKY as a model for anxiety-like disorders when compared to SHR. However, locomotor activity and exploratory behavior of SD was intermediate between SHR and WKY, and SD were similar to WKY in terms of anxiety-like behavior in the elevated-plus maze. These findings do not support the use of WKY as a model of anxiety-like behavior and supports the use of WKY as an appropriate control for SHR as a model for ADHD. Since SD were so similar to WKY, they could be used as an additional control for SHR

## Competing interests

The authors declare that they have no competing interests.

## Authors' contributions

FMH and VAR contributed equally to this work.

LB performed the behavioral studies.
